# Comparison of different treatment planning approaches for intensity-modulated proton therapy with simultaneous integrated boost for pancreatic cancer

**DOI:** 10.1186/s13014-018-1165-0

**Published:** 2018-11-22

**Authors:** Sarah Stefanowicz, Kristin Stützer, Sebastian Zschaeck, Annika Jakobi, Esther G. C. Troost

**Affiliations:** 1 OncoRay – National Center for Radiation Research in Oncology, Faculty of Medicine and University Hospital Carl Gustav Carus, Technische Universität Dresden, Helmholtz-Zentrum Dresden – Rossendorf, Dresden, Germany; 2 Department of Radiotherapy and Radiation Oncology, Faculty of Medicine and University Hospital Carl Gustav Carus, Technische Universität Dresden, Dresden, Germany; 3 Helmholtz-Zentrum Dresden - Rossendorf, Institute of Radiooncology – OncoRay, Dresden, Germany; 4 German Cancer Consortium (DKTK), Partner Site Dresden, and German Cancer Research Center (DKFZ), Heidelberg, Germany; 5 National Center for Tumor Diseases (NCT), Partner Site Dresden, Germany: German Cancer Research Center (DKFZ), Heidelberg, Germany; Faculty of Medicine and University Hospital Carl Gustav Carus, Technische Universität Dresden, Dresden, Germany, and; Helmholtz Association / Helmholtz-Zentrum Dresden - Rossendorf (HZDR), Dresden, Germany; 6 Department of Radiation Oncology, Charité Universitätsmedizin Berlin, Berlin, Germany

**Keywords:** Pancreatic cancer, Intensity modulated proton therapy, Simultaneous integrated boost, Dose escalation

## Abstract

**Background:**

Neoadjuvant radio(chemo)therapy of non-metastasized, borderline resectable or unresectable locally advanced pancreatic cancer is complex and prone to cause side-effects, e.g., in gastrointestinal organs. Intensity-modulated proton therapy (IMPT) enables a high conformity to the targets while simultaneously sparing the normal tissue such that dose-escalation strategies come within reach. In this in silico feasibility study, we compared four IMPT planning strategies including robust multi-field optimization (rMFO) and a simultaneous integrated boost (SIB) for dose-escalation in pancreatic cancer patients.

**Methods:**

For six pancreatic cancer patients referred for adjuvant or primary radiochemotherapy, four rMFO-IMPT-SIB treatment plans each, consisting of two or three (non-)coplanar beam arrangements, were optimized. Dose values for both targets, i.e., the elective clinical target volume [CTV, prescribed dose D_pres_ = 51Gy(RBE)] and the boost target [D_pres_ = 66Gy(RBE)], for the organs at risk as well as target conformity and homogeneity indexes, derived from the dose volume histograms, were statistically compared.

**Results:**

All treatment plans of each strategy fulfilled the prescribed doses to the targets (D_pres(GTV,CTV)_ = 100%, D_95%,(GTV,CTV)_ ≥ 95%, D_2%,(GTV,CTV)_ ≤ 107%). No significant differences for the conformity index were found (*p* > 0.05), however, treatment plans with a three non-coplanar beam strategy were most homogenous to both targets (*p* < 0.045). The median value of all dosimetric results of the large and small bowel as well as for the liver and the spinal cord met the dose constraints with all beam arrangements. Irrespective of the planning strategies, the dose constraint for the duodenum and stomach were not met. Using the three-beam arrangements, the dose to the left kidney could be significant decreased when compared to a two-beam strategy (*p* < 0.045).

**Conclusion:**

Based on our findings we recommend a three-beam configuration with at least one non-coplanar beam for dose-escalated SIB with rMFO-IMPT in advanced pancreatic cancer patients achieving a homogeneous dose distribution in the target while simultaneously minimizing the dose to the organs at risk. Further treatment planning studies on aspects of breathing and organ motion need to be performed.

**Electronic supplementary material:**

The online version of this article (10.1186/s13014-018-1165-0) contains supplementary material, which is available to authorized users.

## Background

Neoadjuvant treatment in non-metastasized, borderline resectable or unresectable locally advanced pancreatic cancer (LAPC) aims at downsizing the tumor achieving a tumor-free resection margin in order to increase both local progression-free and overall survival rates since surgical resection (R0) is the only curative treatment approach in LAPC. In current clinical practice, neoadjuvant chemotherapy with FOLFIRINOX (fluorouracil, leucovorin, irinotecan, and oxaliplatin) is standard of care. In a recent systematic review and patient-level meta-analysis by Suker et al. [1], the median overall survival following FOLFIRINOX was reported to be 24.2 months as opposed to 6–13 months following gemcitabine monotherapy. In the 11 studies reporting outcome measures, the authors noted varying numbers of patients undergoing a subsequent tumor resection or radio(chemo)therapy. Therefore, the authors pledge for a prospective randomized clinical trial addressing the questions on effectiveness and safety of FOLFIRINOX as well as on optimal patient-tailored subsequent treatment.

In the era of three-dimensional conformal radiotherapy (3D-CRT), radiotherapy doses for LAPC patients were hampered by radiosensitive organs at risk (OARs) in proximity of the pancreas thus prohibiting an adequate dose to the target volume. Technical radiation delivery developments in the field of photon-based radiotherapy, i.e., intensity-modulated radiation therapy (IMRT) or stereotactic body radiotherapy (SBRT), enable conformal dose distributions to complex target volumes and, the first, also simultaneous integrated boost (SIB) concepts [2–8]. In the most recent clinical study on dose-escalation to a total dose of 66Gy to the boost target using an IMRT-SIB technique with Tomotherapy, Zschaeck et al. [6] have reported small numbers of patients suffering from acute radiation-induced grade 3 (nausea, abdominal pain and fatigue) or grade 4 (gastrointestinal bleeding) toxicities in a cohort of 28 patients. Meanwhile, results of the prospective phase III PREOPANC study, a randomized, controlled, multicentric superiority trial combining hypofractionated radiotherapy (15 × 2.4Gy) with gemcitabine (1000 mg/m^2^) on days 1, 8, 15, preceded and followed by a modified course of gemcitabine, are eagerly awaited [9].

In recent years charged particles (protons and carbon ions), have been suggested to enable the delivery of a higher radiation dose to the target while at the same time reducing dose to the normal tissues [10]. The physical properties of this alternative radiation modality with a low entrance dose, the maximum dose deposition at the Bragg-Peak, and a steep dose fall-off distant to the Bragg-Peak may further improve the therapeutic possibilities in the anatomical setting of the pancreas with its close-by OARs. Indeed, in the past, different studies reported that a dose-escalated, passively scattered proton therapy (PSPT) of 59.4Gy(RBE) to 70.2Gy(RBE) to the planning target volume (PTV) with concomitant chemotherapy (gemcitabine) enabled a resection for initially borderline resectable pancreatic cancer patients, with favorable survival rates and freedom from local progression [11–15]. A very recent publication on a phase II clinical study (in total 44 patients) combining (4–8 cycles of) neoadjuvant FOLFIRINOX with short-course radiotherapy [5 x 5Gy(RBE) with PSPT (*N* = 15) or 10 x 3Gy with photons (*N* = 12) depending on availability] in clearly resectable disease without vascular involvement or long-course radiotherapy (28 × 1.8Gy with photons; *N* = 17) in the remaining non-metastasized patients, reported remarkable outcome [16]. An R0-resection was achieved in 65% of the evaluable patients with a median progression-free survival of 14.7 months and a 2-year overall survival of 56%. Only 6% of the patients experienced an isolated locoregional recurrence as the initial site of treatment failure.

In previously published comparative treatment planning studies for PSPT and pencil-beam scanning (PBS) for treatment of pancreatic cancer, the dosimetric advantage of proton therapy over photons could be shown. However, varying numbers and directions of the applied fields were used due to the complex abdominal anatomy and the lack of consensus guidelines [17–23]. In order to reduce dose to the OARs and enabling sufficient dose to the complex target of the pancreas and elective lymph nodes, intensity-modulated proton therapy (IMPT) with PBS is of great advantage. Moreover, multi-field optimization (MFO) for IMPT provides a high degree of dose modulation by optimizing all spots and their energies of each field taking into account the OAR dose constraints. One of the major challenges in proton beam therapy, in particular of pencil beam scanning, is its high sensitivity to changing anatomy caused by, i.e., organ motion, density changes, and positioning errors. However, to tackle these uncertainties, the number of beams, the beam direction, and robust treatment planning algorithms can improve the robustness of an IMPT plan. Robust treatment planning algorithms take into account setup and density uncertainties resulting from setup errors due to patient positioning or from converting the computed tomography (CT) number into stopping power ratios, respectively [24, 25]. Thus, the proton treatment technique as well as the beam directions should both be chosen cautiously.

The aim of this retrospective, in silico treatment planning study was to prove the feasibility of robust multi-field optimized IMPT (rMFO-IMPT) treatment planning for the SIB technique with dose escalation in the gross tumor volume in pancreas while meeting the OARs dose constraints.

## Methods

### Patient and tumor characteristics

Six patients with a non-resectable LAPC or locally recurrent pancreatic cancer (LRPC) having received primary or adjuvant radiochemotherapy with Tomotherapy-based photon therapy at the Charité Universitätsmedizin Berlin were selected for this comparison (Table 1) [6]. For each patient, a free-breathing treatment planning CT (Sensation Open, Siemens Healthineers, Erlangen, Germany) in supine position had been acquired with a 2 mm slice thickness.

**Table 1:** Patient, tumor and treatment characteristics

Patient	Gender	Primary tumor location	TN Classification	Treatment intent	Resection of duodenum	Volume [ccm]		OARs *within* or immediately adjacent to targets
						GTV	CTV	
1	M	head	pT3 pN0	primary	yes	25.4	204.4	*Small bowel, large bowel, liver,* left kidney
2	M	head	pT2 pN0	primary	yes	89.9	286.3	*Small bowel*
3	F	head	cT3 cN0	primary	yes	58.2	144.2	*Small bowel, liver*
4	M	body	cT4 cN1	primary	no	123.0	356.7	*Duodenum, small bowel, liver,* large bowel
5	M	body + tail	pT3 pN0	adjuvant (individualized treatment)	no	39.0	577.3	*Duodenum, small bowel, liver, stomach,* large bowel
6	M	body	cT4 cN1	primary	no	90.7	197.7	*Duodenum, small bowel, stomach,* large bowel

### Dose prescription and treatment planning

On the treatment planning CT, the gross tumor volume (GTV), serving as target volume for the boost, consisted of the primary or recurrent tumor, and the CTV included the putative microscopic tumor extension and the regional lymph nodes [6]. Furthermore, the following OARs were contoured: spinal cord, liver, right and left kidney, stomach, duodenum, small bowel and large bowel. In all patients, the OARs overlapped with the GTV and/or CTV (Table 1) resulting in careful balancing of the maximum tolerable dose versus coverage of the target volume (no planning risk or integrated protection volumes were generated). For each patient, four different rMFO-IMPT plans with a SIB were generated using the treatment planning system RayStation Research V5.99 (RaySearch Laboratories AB, Stockholm, Sweden). The treatment plans were optimized to deliver at least 95% of the prescribed doses (D_pres(GTV, CTV)_ = 100% relative dose) of 66Gy(RBE) and 51Gy(RBE) to 95% of the GTV and the CTV (D_95%_ ≥ 95%), respectively. The near dose maximum in 2% of the volume (D_2%_) was not to exceed 107% of the D_pres_ in each target. The plan objectives and weights in the plan optimizer for the OARs were chosen taking into account the institutional guidelines and QUANTEC dose constraints as summarized in Table 2 [26]. It has to be mentioned that reduction of the dose to the overlapping OARs such as duodenum and parts of the stomach and the small bowel were of less priority due to the pancreatoduodenectomy after radiotherapy. To guide the dose fall-off from the GTV to the CTV within a range of 10 mm, an auxiliary ring structure (GTV_10mm_; Additional file 1: Figure S1) was used. A further auxiliary structure termed CTV_eval_, defined as the CTV minus the GTV and the GTV_10mm_ [CTV_eval_ = CTV - (GTV + GTV_10mm_)], was created in order to lead the optimizer to a homogeneous dose distribution to this remaining CTV and to exclude the high dose gradient volume for evaluation. Several auxiliary structures were used to reduce the dose to the OARs and to avoid hot spots outside the target volumes. Furthermore, more than 100 iterations were performed for an adequate plan optimization using the pencil beam algorithm. Since a CTV-based treatment planning concept was used, robust optimization was applied to account for a random setup uncertainties of 3 mm in each orthogonal direction and a systematic range uncertainties of 3.5% in the optimization for both target volumes. In all treatment plans the D_mean_ of the boost target (GTV) was normalized to D_pres_ = 66 Gy(RBE).

**Table 2:** Dose constraints for the organs at risk adhering to the local guidelines and QUANTEC [26]

OAR	Dose constraint
Spinal cord	D_max_ ≤ 45 Gy
Liver	D_mean_ ≤ 30 Gy V_30Gy_ ≤ 30%
Kidney	D_mean_ ≤ 18 Gy V_12Gy_ ≤ 55% V_20Gy_ ≤ 32%
Stomach	D_max_ ≤ 54 Gy V_50Gy_ ≤ 2% V_45Gy_ ≤ 25%
Duodenum	D_max_ ≤ 55 Gy V_45Gy_ ≤ 25%
Large Bowel	V_15Gy_ ≤ 120 ccm
Small Bowel	V_15Gy_ ≤ 120 ccm

*Abbreviations*: *D*_*max*_ maximum dose, *D*_*mean*_ mean dose, *V*_*xGy*_ volume receiving x-Gy

Four different field-setups were generated, while beam angles were chosen individually taking into account the patient’s anatomy [27]:

- (S1) two posterior oblique beams,
- (S2) a lateral right beam and a left posterior oblique beam,
- (S3) two oblique posterior beams plus a right-sided non-coplanar beam, and
- (S4) three non-coplanar beams from posterior and the right side.

All beams were weighted by the optimization algorithm.

For the dose calculation, the beam model of the IBA universal nozzle of the University ProtonTherapy Dresden without a range shifter was used. The distance of the nozzle to the gantry isocenter was fixed to 50 cm. The spot size sigma (in air) of the pencil beam ranged from 4 mm for 230 MeV to 8 mm for 100 MeV. The dose distribution, calculated in a dose grid of 3 mm × 3 mm × 3 mm with the pencil beam algorithm, was a superposition of all pencil beam spots of protons with a RBE of 1.1, whereas positions and spot distances were set by the treatment planning system.

### Treatment plan evaluation

For each patient and planning strategy, the dose distribution and the dose volume histograms (DVH) of the targets and the OARs were approved and deemed clinically acceptable by a radiation oncologist based on the predefined dose constraints, as listed in Table 2, using the RayStation evaluation tool. Due to varying ratios of the CTV-GTV and the CTV_eval_, the dose distributions of both structures were evaluated (Additional file 1). Furthermore, Paddick’s conformity index of both target volumes, GTV and CTV, were calculated [28]. The homogeneity index (HI), defined as (D_5%_-D_95%_/D_pres_) × 100, was determined for the GTV and the CTV_eval_.

In order to prove the robustness of the targets, the dose distributions of eight possible scenarios were calculated based on the nominal treatment plan considering displacements of the patient in each orthogonal direction (x,y,z = ±3 mm) and density errors of ±3.5%. We defined a treatment plan as robust if the dose coverage in each scenario met at least the target dose constraints of D_95%_ ≥ 95% and D_2%_ ≤ 107%.

### Statistical analyses

The non-parametric *Friedman* test (*α* = 0.05*,* significance at *p* < 0.05) was performed to detect statistically significant differences among the four strategies regarding the median values for doses to targets and OARs, or target CI and HI. In case of statistical significance, the respective median values were compared using the non-parametric post hoc test of *Dunn* with a *Bonferroni* correction (*α* = 0.05*,* significance at *p* < 0.05). Statistical analyses were performed using IBM® SPSS® Statistics (Version 25.0.0.1, IBM Corp., Armonk, NY, USA). For subgroup analysis of less than six patients, no statistical evaluation of dose values was performed.

## Results

**Fig. 1:**
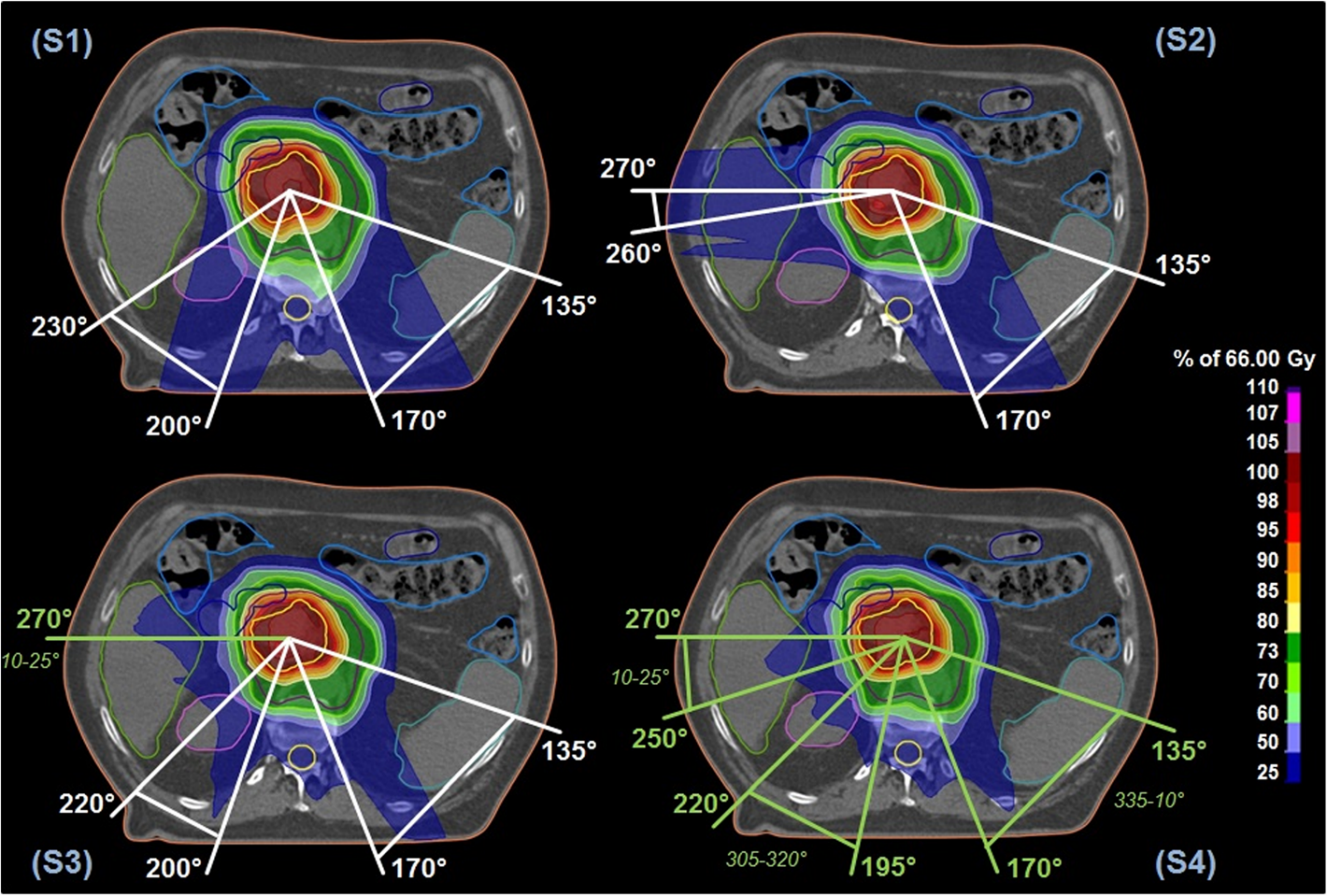
Overview of the beam configurations and resulting dose distribution of the four different treatment planning strategies (S1-S4) evaluated in this in silico treatment planning study. Coplanar beam directions are marked in white, non-coplanar beam directions in green. Moreover, the range of applied beam and couch angles in the six patients is given per beam direction. For patient 2, the resulting dose distribution to the CTV (violet) and GTV (orange) is shown as color wash superimposed on the planning CT

The results of the patient individual beam angles of the four different planning strategies are shown in Fig. 1 and in Additional file 2. The beam directions were chosen irrespective of the primary target location in the pancreatic head or tail.

**Fig. 2:**
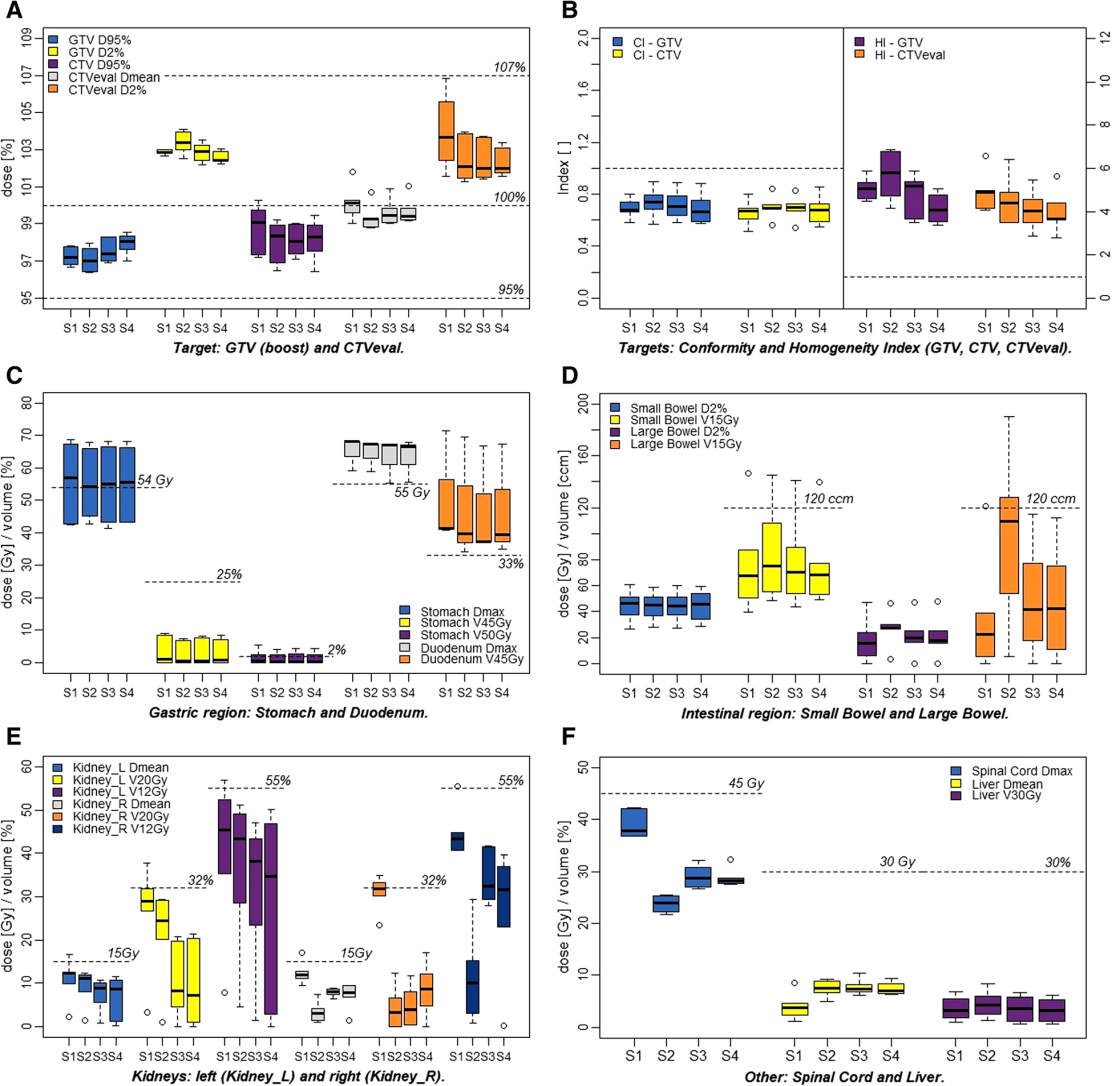
Box-and-whisker-plots showing the dose parameters of the targets (**a, b**) and the organs at risk (**c-f**) of all treatment plans sorted by the treatment planning strategies (S1)-(S4). Dose constraints are marked with dashed lines, and statistically significant findings are listed in the Additional file 3 (Abbreviations: Dmean: mean dose; Dmax: maximum dose; VxGy: volume receiving x-Gy; D2%: near dose maximum, dose received by 2% of the volume; CI: conformity index; HI: homogeneity index)

All treatment plans fulfilled the prescription dose requirements of D_mean_, D_95%_ and D_2%_ to the GTV as well as D_95%_ to the CTV and to the CTV-GTV (Fig. 2a, Additional file 2). The D_mean_ and D_2%_ dose constraints were met for the CTV_eval_, however, the D_2%_ of the CTV-GTV always exceeded the preset dose value of 107% due to the dose gradient (D_2%_ > 125.9% of 51Gy, Additional file 2). Comparing the four strategies in terms of median values to the GTVs, the D_95%_ and D_2_ of S2 were lowest and highest, respectively, in particular when compared to S4 (*p* = 0.002 and *p* = 0.01, Additional file 3). The median D_mean_ to the CTV_eval_ varied between the four beam configurations, but only with a significant difference between S1 and S2 (*p* = 0.005). The median CI of the dose distribution to the GTV (S1: 0.68, S2: 0.74, S3: 0.70, S4: 0.66) and the CTV (S1: 0.68, S2: 0.74, S3: 0.70, S4: 0.66) were similar among the four planning strategies (*p* = 0.09 and *p* = 0.102, Fig. 2b, Additional file 2, Additional file 3). The median HI of the GTV statistically significantly differed between S2 and S4 (*p* = 0.002) and between S1 and S3 or S4 (*p* = 0.044 and *p* = 0.01).

Depending on the chosen beam combination, the doses to the OARs differed for the four treatment strategies (Table 3, Fig. 2). For the stomach and duodenum none of the beam configurations were favorable in terms of lowest dose distributions to these OARs. Since half of the patients had undergone a pancreatoduodenectomy, the dose distribution to the duodenum could only be evaluated for three patients (Table 1). In these patients, the D_max_ and the V_45Gy_ dose constraints were not met for any of the in silico treatment plans, respectively. For the stomach the median D_max_ per beam configuration was also exceeded, but the median results of each strategies were within the constraints for the near maximum dose D_2%_ and the volume parameters V_45Gy_ and V_50Gy_ (Table 3, Fig. 2c). Slightly increased doses to the stomach were found for targets located within the pancreatic body (Additional file 4).

**Table 3:** Dose parameters to organs at risk for each patient (1–6) and planning strategy (S1-S4) given per patient and as a cohort median

	Organs at risk																					
	No	Stomach				Duodenum			Small Bowel		Large Bowel		Spinal Cord		Liver		Left Kidney			Right Kidney		
		D_max_ [Gy]	D_2%_ [Gy]	V_45Gy_ [%]	V_50Gy_ [%]	D_max_ [Gy]	D_2%_ [Gy]	V_45Gy_ [%]	D_2%_ [Gy]	V_15Gy_ [ccm]	D_2%_ [Gy]	V_15Gy_ [ccm]	D_max_ [Gy]	D_2%_ [Gy]	D_mean_ [Gy]	V_30Gy_ [%]	D_mean_ [Gy]	V_12Gy_ [%]	V_20Gy_ [%]	D_mean_ [Gy]	V_12Gy_ [%]	V_20Gy_ [%]
(S1)	1	49.8	29.3	0.1	0.0	–	–	–	51.6	146.6	46.9	121.1	37.3	26.7	8.5	6.9	12.3	52.3	30.8	9.4	40.6	23.5
	2	42.6	18.7	0.0	0.0	–	–	–	42.1	58.1	14.6	10.2	36.9	33.9	1.2	1.0	2.2	7.8	3.3	11.7	42.4	30.2
	3	42.6	19.4	0.0	0.0	–	–	–	51.1	87.8	0.1	0.0	42.3	36.0	4.6	5.5	12.2	44.7	31.9	12.7	44.3	33.3
	4	64.4	43.7	1.8	1.1	68.2	63.3	71.5	26.7	39.6	24.1	34.3	36.8	33.5	2.4	2.1	9.8	35.4	26.7	11.1	40.6	31.4
	5	68.9	50.6	8.5	2.5	59.1	51.8	40.8	37.3	77.7	6.2	5.6	42.1	37.8	4.0	4.4	12.6	46.0	27.0	12.1	44.8	32.3
	6	67.3	57.3	9.0	5.5	68.1	63.4	41.4	60.9	50.4	16.3	39.3	38.3	30.8	3.5	1.8	16.5	57.0	37.7	17.1	55.5	35.0
Median		57.1	36.5	1.0	0.5	68.1	63.3	41.4	46.6	67.9	15.5	22.3	37.8	33.7	3.8	3.3	12.3	45.4	28.9	11.9	43.4	31.9
(S2)	1	48.3	32.8	0.1	0.0	–	–	–	51.5	145.0	46.6	128.0	21.7	16.1	9.0	8.4	12.0	51.2	29.5	4.0	15.1	6.6
	2	42.6	19.9	0.0	0.0	–	–	–	40.8	75.0	27.0	53.8	24.6	21.1	4.9	1.3	1.5	4.5	1.0	7.4	29.4	12.4
	3	45.2	17.2	0.0	0.0	–	–	–	50.1	108.2	3.6	5.7	25.4	23.0	7.8	6.0	10.7	41.7	23.9	1.1	0.8	0.0
	4	60.4	38.3	1.0	0.4	67.3	62.5	69.5	28.0	48.5	29.8	190.5	25.4	22.3	6.6	2.5	8.1	28.6	20.2	2.2	5.6	0.3
	5	68.0	50.4	6.7	2.3	58.9	52.1	34.1	36.8	75.0	26.9	99.5	23.1	21.8	7.2	4.6	11.5	44.9	25.2	4.1	14.5	6.4
	6	65.9	55.1	7.4	4.2	67.3	63.9	39.8	59.1	55.6	27.8	119.7	22.2	19.4	9.2	4.0	12.3	49.1	29.1	1.4	3.1	0.0
Median		54.4	35.6	0.5	0.2	67.3	62.5	39.8	45.4	75.0	27.4	109.6	23.9	21.5	7.5	4.3	11.1	43.3	24.5	3.1	10.0	3.4
(S3)	1	49.3	30.7	0.1	0.0	–	–	–	51.5	141.2	47.1	114.8	27.0	20.1	10.5	6.7	9.8	47.1	10.2	6.3	27.9	0.5
	2	43.4	17.6	0.0	0.0	–	–	–	39.3	61.7	19.0	17.8	28.4	24.8	6.1	1.2	0.8	1.4	0.0	8.6	35.1	11.6
	3	41.3	18.3	0.0	0.0	–	–	–	49.9	89.9	0.1	0.0	32.2	28.5	8.3	5.8	7.8	33.8	6.3	7.3	30.0	3.1
	4	61.0	40.3	1.2	0.5	67.2	62.5	66.7	27.6	43.6	25.6	77.3	26.8	24.1	7.2	2.4	5.6	23.4	4.5	8.8	41.5	4.5
	5	68.3	50.6	7.5	2.6	55.2	51.7	37.2	37.8	79.6	16.1	18.2	29.2	25.7	7.6	4.6	10.0	42.6	20.8	8.4	41.8	8.0
	6	66.5	55.2	8.2	4.5	67.5	64.7	37.4	60.0	54.0	21.0	64.9	30.9	27.6	6.8	0.7	10.6	43.3	19.7	7.6	29.3	0.4
Median		55.2	35.5	0.6	0.3	67.2	62.5	37.3	44.6	70.7	20.0	41.5	28.8	25.3	7.4	3.5	8.8	38.2	8.3	8.0	32.6	3.8
(S4)	1	50.5	31.7	0.3	0.0	–	–	–	51.7	140.0	47.8	112.6	32.3	28.0	9.4	6.2	10.6	50.1	7.8	8.2	34.6	12.0
	2	43.2	20.7	0.0	0.0	–	–	–	40.4	60.1	15.5	11.0	28.7	25.2	6.3	1.1	0.2	0.0	0.0	9.5	37.0	17.1
	3	43.3	16.8	0.0	0.0	–	–	–	53.8	77.5	0.1	0.0	28.8	25.9	8.4	5.3	6.6	25.6	6.5	6.8	23.1	4.7
	4	60.6	41.4	1.3	0.6	66.4	62.6	67.3	28.6	49.2	25.0	75.5	27.7	25.4	7.0	2.3	1.2	2.9	1.0	9.4	39.6	6.5
	5	68.3	50.3	7.2	2.3	55.6	51.5	35.0	34.4	77.3	15.7	18.9	27.5	24.4	6.5	4.1	11.5	46.8	21.3	7.4	28.8	10.9
	6	66.2	55.0	8.4	4.3	67.9	64.0	39.4	59.7	53.6	19.2	66.5	27.8	24.9	7.0	0.6	10.7	43.6	20.3	1.4	0.3	0.0
Median		55.6	36.5	0.8	0.3	66.4	62.6	39.4	46.0	68.7	17.5	42.7	28.2	25.3	7.0	3.2	8.6	34.6	7.1	7.8	31.7	8.7

*Abbreviations*: *D*_*max*_ maximum dose, *D*_*mean*_ mean dose, *V*_*xGy*_ volume receiving x-Gy, *D*_*2%*_ near dose maximum; dose received by 2% of the volume

Although the large and small bowel were located within or immediately adjacent to the GTV and CTV, illustrated by the high D_2%_ values, the median V_15Gy_ of the small and large bowel met the pre-specified dose constraint (Fig. 2d). While the median volumes of V_15Gy_ ≤ 120ccm for the small bowel were similar for all beam combinations, a statistically significantly increased median value for the large bowel was found for S2 compared to S1 (*p* = 0.005). Furthermore, the median irradiated volume to the large bowel for tumors within the pancreatic body were higher for all strategies (Additional file 4: E). Noteworthy, the median irradiated volumes of the small bowel were not influenced by the different treatment strategies for the subgroup of the pancreatic body, whereas for the pancreatic head S4 reduced those median irradiated volumes when compared to the other strategies (Additional file 4: E).

The median D_mean_, V_12Gy_ and V_20Gy_ to the kidneys were met by all treatment planning strategies (Fig. 2e). For the left kidney, the median values of these dose constraints were statistically significant lower for the three-beam strategies (S3 and S4) compared to the two-beam posterior-oblique strategy (S1, *p* < 0.045). For the right kidney, S2 resulted in statistically significantly reduced doses to all dose constraints when compared to S1 (*p <* 0.004), even though S3 and S4 also spared the radiation dose to the right kidney well (not significant).

Regarding the spinal cord, the D_max_ constraint (as well as the D_2%_) was met by each planning strategy, albeit that the median D_max_ of S2 was statistically significantly lower compared to the other strategies (*p* < 0.001, Fig. 2f), respectively. The median D_mean_ and the V_30Gy_ of the liver were within limits for all strategies, with the lowest median dose value for S1 (Fig. 2f).

The robustness of the coverage of the CTV was reached for all treatment plan of each strategy (Additional file 5: E-H). Single scenarios did not fulfill the robustness constraint D_95%_ for the GTV and D_2%_ for the CTV_eval_ (Additional file 5: A-D), however, the dosimetric values were all close to the minimum volume level for the coverage.

## Discussion

In our feasibility study we compared dosimetric parameters of four different robust multi-field optimized IMPT-SIB strategies for dose escalation to 66Gy(RBE) in locally advanced pancreatic cancer patients scheduled to undergo adjuvant or primary radio(chemo)therapy. The results show that treatment planning using a robust, multi-field optimized proton technique with simultaneous integrated boost is possible using a two-beam- or a three-beam-configuration. While the preset dose prescriptions for the GTV as well as the CTV were reached by all strategies, sparing of the OAR depended on the number of beams chosen as well as on the primary tumor location.

For radiation treatment of pancreatic cancer, treatment planning of a SIB with an escalated dose inside the boost using rMFO-IMPT is highly challenging for several reasons:

First, the sizes of both target volumes, i.e., GTV and CTV, were small (median GTV: 74.1 ccm [range: 25.0–123.0 ccm], median CTV: 245.4 ccm [range: 144.2–356.7 ccm]; see Table 1) and their interdependence large in comparison to IMPT-SIB treatment plans in head-and-neck cancer patients [i.e., median CTV1: 152.5 ccm (range: 96.8–20.6 ccm), median CTV2: 264.9 ccm (range: 218.5–426.7ccm), median CTV3: 220.2ccm (141.8–282.3ccm); [29]]. Consequently, the relative target coverage is more sensitive to under- or over-exposed volumes, even for the dose coverage (D_95%_). Despite the steep distal dose gradient of the proton beams and the usage of auxiliary structures, reducing the distance of the high dose gradient between both targets is limited when maintaining the robustness of the treatment plans. Thus, if the distance between the GTV and CTV was small (< 10 mm), the dose gradient reached into the normal tissue in five of the six cases causing high dose regions [> 51Gy(RBE)] in the close-by OARs. For our study, we used a setup uncertainty value of 3 mm assuming an image-guided clinical workaround. Nevertheless, it cannot be ruled out that an increased setup uncertainty value, i.e., of 5 mm, may generate an enlarged D_95%_ volume around the target volumes resulting in increased dose to the OARs in direct proximity and in an expanded high-dose gradient region. Balancing the gradient and the robustness also has taken into account in the robustness evaluation. Single scenarios of the GTV did not reach the D_95%_ constraint, however, they are very close to the minimum dose coverage level. Since they occurred when a perturbed dose distribution with a setup uncertainty was calculated, they can be disregarded. Such random uncertainties will be smeared out after all fractions. The D_2%_ of the CTV_eval_ also has to evaluate with care since the dose gradient in the different scenarios may be expanded into the CTV_eval_.

Second, the literature on proton beam therapy for pancreatic cancer differs regarding the number of beams and beam directions due to the lack of consensus guidelines. Commonly, the usage of two or three coplanar beams is preferred depending on the treatment planning modality (passive scattering or active scanning) [17–23]. In our in silico treatment planning feasibility study on rMFO-IMPT for a dose-escalated SIB, the number of beams and their directions in S1-S4 were cautiously chosen based on the experience of the aforementioned studies to keep the dose to radiation-sensitive organs at risk as low as possible and to ensure a homogenous and conform dose coverage of both targets. Despite a possible improvement in the target conformity, the low dose to the OARs in the beam entrance and, in the robustness of the dose distribution, more than three beams are not advisable considering the complex anatomy of the close-by OARs and the impact of inhomogeneities, i.e., the continuously gas movement of the bowel, to the range of the protons [30].

A two-beam arrangement with at least one beam from the anterior direction may reduce the dose to the kidneys, however, this beam direction does not take into account the impact of the continuously changing filling of the bowel and motion of the abdominal wall to the proton range [17–19, 30]. Therefore, we employed a combination of two oblique beams (S1), particularly sparing the bowel and reported to be robust against inter-fractional motion in carbon ions, and a combination of one left oblique and one right lateral beam (S2) in order to minimize the dose to the kidneys [21, 31]. Here, S1 resulted in the lowest dose to the large bowel and the liver. However, the doses to both kidneys and to the spinal cord were rather high almost reaching the maximum dose constraint. S2 offered the possibility to spare one kidney completely, but conversely, this beam arrangement may result in clinically relevant doses to the large bowel. Compared to IMRT and PBS-IMPT treatment plans (left lateral oblique, posterior oblique) reported by Ding et al. [21] with a D_pres_ of 50.4Gy to the PTV (without a SIB) our dose to the kidneys and the small bowel were mainly lower, e.g., small bowel V_15Gy_: 269.5ccm (IMRT) vs. 174.2ccm (PBS) vs. 67.9ccm (S1) vs. 75.0ccm (S2).

Moreover, we investigated two three-beam configurations. Although more than two beams increase the low dose volume in the normal tissue surrounding the target, the strategies S3 and S4 offer more degrees of freedom to reduce the dose to the normal tissue to clinically accepted values and to cover the complex targets more homogeneously. Consequently, S3 and S4 spare the bowel while simultaneously keeping the dose to the remaining OARs low. Nichols et al. [20] dosimetrically compared IMRT and a PSPT plans, the latter with two oblique posterior fields and one left lateral field, to a prescribed dose of 50.4Gy to the boost (PTV: 45Gy) and reported a statistically significant reduction of radiation dose to the right kidney, the small bowel and the stomach in the PSPT plans. Taking into account the previous experience using non-coplanar beams for IMRT plans, we chose a non-coplanar right lateral direction (S3) attempting to reduce the dose to the gastrointestinal organs [32]. The last beam arrangement (S4) was based on previous work by Thompson et al. [22], the first to compare IMRT plans with PSPT and PBS treatment plans, using three non-coplanar proton beams (D_pres,PTV_ = 55Gy, gantry: ~ 160°, ~ 170°, ~ 215° with unknown coach angle). With this beam approach, Thompson et al. [22] compared to Nichols et al. [20] showed a reduction of the dose to the small bowel (V_20Gy_: 9.8% vs. 15.4%, V_45Gy_: 4.2% vs. 8.4%) with a non-clinically relevant dose increase to the stomach (V_20Gy_: 11.1% vs. 2.3%, V_45Gy_: 5.8% vs. 0.1%) despite a higher prescribed dose to the target compared to the first. When using rMFO-IMPT with SIB and even prescribing a higher dose to the boost, our results were even lower than those by Thompson et al. [22] (small bowel: V_20Gy_ = 6.2%, V_45Gy_ = 2.5%; stomach: V_20Gy_ = 6.4%, V_45Gy_ = 0.8%). Counter intuitively, the positive effect of utilizing non-coplanar beams for a dose reduction to the gastrointestinal organs was only found for the small bowel if the tumor was located in the head of the pancreas (Additional file 4: E).

To summarize, each beam configuration has several dosimetric advantages and disadvantages. However, the three-beam configurations are of clinical relevance showing the potential to reduce the normal tissue complication probability of the OARs in an intensified treatment while increasing the homogeneity of the dose distribution. The comparison to PTV-based proton and photon treatment plans is certainly not precise, however, CTV-based, robust optimized photon and proton treatment planning studies are missing for pancreatic cancer in the literature.

Third, the proximity or overlap of the OARs, i.e., duodenum and stomach, with the target volumes is a major issue in treatment planning for pancreatic cancer. Bouchard et al. [33] postulated a required distance of approximately 20 mm between the OARs and the GTV for safe dose escalation to 72Gy(RBE) with PSPT for pancreatic target volumes. Due to the complex abdominal anatomy, this distance is rarely applicable to tumors in the pancreatic region. Thus, overlapping or immediately surrounding structures are difficult to protect. To accomplish this, Brunner et al. [34] suggested a simultaneous integrated protection (SIP) area, which contains the intersection volume of the OARs with the target volumes, reducing the dose within the SIP to the respective dose constraint of the affected OAR. Although this method was proposed for IMRT, this method should also be tested for IMPT in further studies.

Fourth, the non-coplanar beam configuration faces technical challenges. The couch angles are limited by the construction of the nozzle and the distance to the isocenter. Furthermore, the CT images need to be of sufficient length to allow a correct dose calculation of the treatment plan with non-coplanar beams. It needs to be considered that a non-coplanar beam could extend the path of the beam through the patient leading to potential uncertainties of the proton range. Lastly, non-coplanar treatment setups are more difficult for the treatment planner during the treatment planning process (e.g., spatial aptitude, sources for collision) and for the radiation treatment technologists during the actual irradiation (e.g., collisions, time). Thus, non-coplanar beams should only be used if the benefit of sparing normal tissue and avoidance of density inhomogeneities is increased.

Finally, the quality of treatment plans depends on the experience of the treatment planner, the treatment technique and the optimization algorithm. In our study we used an objective weighted optimization for IMPT, of which the results are systematically influenced by the interaction of each objective weight chosen by the treatment planner. Thus, it may well be that a better dose distribution may be reached, e.g., by a multi-criteria optimization algorithm. Furthermore, due to the computation time of robust optimization with a small voxel resolution in a Monte Carlo algorithm, we decided to use a dose calculation grid with an acceptable resolution as well as the pencil beam algorithm for this study. Considering large homogeneity differences as well as the proximity of radiation-sensitive abdominal organs, a robust Monte Carlo optimization and an adequate grid resolution are recommended for clinical proton therapy plans.

Apart from the above stated some limitations of our presented study need to be taken into account. Since only a limited number of patients was investigated in this in silico treatment planning study, further studies need to confirm our findings and to verify the results for each tumor location group, in particular for the duodenum. Four-dimensional CT scans were not available for this retrospective study, since they were not acquired for clinical routine in Tomotherapy. Hence, the intrafractional movement of the target and the OARs could not be considered although this is an important issue in proton therapy. Breathing can result in undesirable over- and undershooting in target volumes (interplay effect) or even in increased dose to the normal tissue [35, 36]. Using dose escalation approaches, the impact of intra- as well as interfractional organ motion is of highly clinical relevance. To overcome this, additional treatment planning studies are underway to estimate the influence of breathing and organ motion on the dose distribution and the consequential robustness of the treatment plans.

## Conclusion

Disregarding the influence of inter- and intrafractional organ motion on the dose distribution, simultaneous dose escalation to the high dose volume is feasible using rMFO-IMPT treatment strategies of two or three beams. Based on our findings, we recommend a three-beam configuration with at least one non-coplanar beam for rMFO-IMPT-SIB in advanced pancreatic cancer patients achieving a homogeneous dose distribution in the target while simultaneously minimizing the dose to the organs at risk. Further studies on the influence of the interplay effect on the dose distribution in dose-escalated SIB strategies have to be performed.

## Additional files

Additional file 1: Illustration of the targets and the auxiliary structures. (PDF 311 kb)

Additional file 2: Table of beam parameters and doses to the target. (PDF 547 kb)

Additional file 3: Statistical analysis. (PDF 511 kb)

Additional file 4: Subgroup analysis. (PDF 487 kb)

Additional file 5: Robustness evaluation. (PDF 415 kb)

## Abbreviations

(4D-)CT: (4-dimensional) computed tomography
ccm: Cubic centimeter
CTV: Clinical target volume
D_2%_: Near dose maximum; dose received by 2% of the volume
D_max_: Maximum dose
D_mean_: Mean dose
D_pres_: Prescribed dose
D_X%_: Dose received by X% of the volume
GTV: Gross tumor volume
Gy: Gray
IMPT: Intensity-modulated proton therapy
IMRT: Intensity-modulated radiotherapy (with photons)
LAPC: Locally advanced pancreatic cancer
OAR: Organ at risk
PBS: Active pencil beam scanning
PSPT: Passive scattering proton therapy
PTV: Planning target volume
*p*-value: Probability value
RBE: Relative biological effectiveness
rMFO: Robust multi-field optimization
S1: Strategy 1, etc.
SIB: Simultaneous integrated boost
vs.: Versus
V_XGy_: Volume receiving x-Gy
