# Supplementary material for: Comparison of different treatment planning approaches for intensity-modulated proton therapy with simultaneous integrated boost for pancreatic cancer
Source: Radiat Oncol. 2018 Nov 22;13:228. doi: 10.1186/s13014-018-1165-0 (PMC6249773; doi:10.1186/s13014-018-1165-0)
Supplement: Supplementary file 1 — Illustration of the targets and the auxiliary structures. (PDF 311 kb) [file 13014_2018_1165_MOESM1_ESM.pdf]

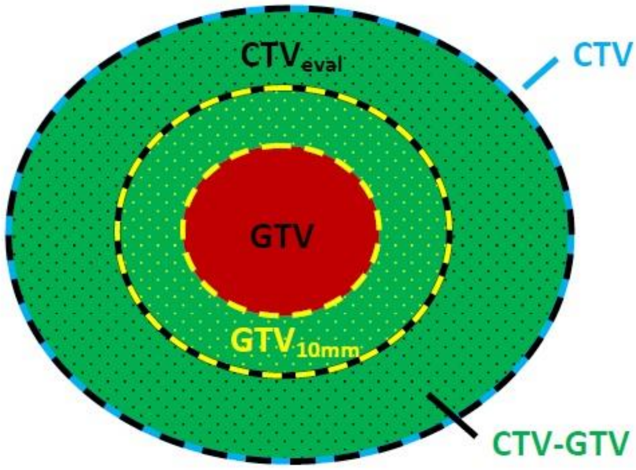

Additional file 1: Illustration of the targets and the auxiliary structures. The boost is defined as the gross tumor volume (GTV; solid red), the volume covering the microscopic tumor extension and elective lymph nodes as clinical target volume (CTV; dashed blue). The green region presents the CTV without the GTV (CTV-GTV). In order to guide the dose fall-off, a ring structure of 10 mm around the GTV was created (GTV_{10mm}, yellow dashed line and region) and a homogeneous dose was allocated to the CTV beyond the GTV_{10mm} [CTV_{eval}, black dashed line and region; CTV – (GTV + GTV_{10mm})].
