# Supplementary material for: Comparison of different treatment planning approaches for intensity-modulated proton therapy with simultaneous integrated boost for pancreatic cancer
Source: Radiat Oncol. 2018 Nov 22;13:228. doi: 10.1186/s13014-018-1165-0 (PMC6249773; doi:10.1186/s13014-018-1165-0)
Supplement: Supplementary file 2 — Table of beam parameters and doses to the target. (PDF 547 kb) [file 13014_2018_1165_MOESM2_ESM.pdf]

Additional file 2: Beam parameters and doses to the targets for each patient (1-6) and planning strategy (S1-S4), given per patient and as a cohort median.

		Beam parameter			Target volumes												
		Beam/couch angle			GTV		CTV		CTV-GTV		CTV_{eval}			Conformity and homogeneity index			
	No.	Beam 1 [°]	Beam 2 [°]	Beam 3 [°]	$D_{95\%}$ [%]	$D_{2\%}$ [%]	D_{mean} [Gy]	$D_{95\%}$ [%]	$D_{95\%}$ [%]	$D_{2\%}$ [%]	D_{mean} [Gy]	$D_{95\%}$ [%]	$D_{2\%}$ [%]	GTV CI [ ]	GTV HI [ ]	CTV CI [ ]	CTV_{eval} HI [ ]
(S1)	1	135/0	220/0	-	96.7	103.0	52.6	97.2	97.1	127.8	50.5	96.8	101.6	0.80	5.87	0.69	4.17
	2	150/0	200/0	-	97.5	102.6	56.0	98.9	98.6	129.9	51.1	98.0	103.9	0.66	4.74	0.65	4.95
	3	150/0	200/	-	97.8	103.0	58.7	99.7	99.2	130.8	50.8	97.5	102.4	0.58	4.59	0.61	4.08
	4	155/0	205/0	-	96.9	102.9	54.9	100.3	100.0	126.8	51.9	99.7	105.6	0.66	5.34	0.69	4.82
	5	160/0	220/0	-	97.8	102.8	52.7	97.3	97.3	127.8	51.0	97.0	106.8	0.74	4.46	0.80	6.55
	6	170/0	230/0	-	96.8	102.8	56.6	99.2	98.7	130.6	51.1	97.9	103.5	0.69	5.34	0.51	4.98
**Median**					**97.2**	**102.8**	**55.3**	**99.1**	**98.5**	**128.8**	**51.1**	**97.7**	**103.7**	**0.68**	**5.04**	**0.67**	**4.89**
(S2)	1	135/0	260/0	-	96.4	104.1	52.6	96.9	96.8	128.2	50.4	96.6	101.5	0.90	6.86	0.69	4.30
	2	150/0	270/0	-	97.2	103.4	55.6	98.0	97.7	129.7	50.6	96.9	102.5	0.69	5.62	0.70	4.89
	3	150/0	270/0	-	96.4	104.0	57.6	98.9	98.2	130.2	50.4	97.3	101.3	0.57	6.77	0.68	3.47
	4	155/0	270/0	-	96.8	103.4	54.1	99.2	98.9	125.9	51.4	98.5	104.0	0.70	5.98	0.72	4.50
	5	160/0	270/0	-	98.0	103.0	52.4	96.5	96.4	128.4	50.6	96.0	103.9	0.78	4.72	0.84	6.39
	6	170/0	270/0	-	98.0	102.5	56.5	98.7	98.3	129.8	50.6	97.6	101.6	0.80	4.13	0.56	3.47
**Median**					**97.0**	**103.4**	**54.8**	**98.3**	**97.7**	**129.0**	**50.6**	**97.1**	**102.1**	**0.74**	**5.80**	**0.69**	**4.40**

	No.	Beam/couch angle			GTV		CTV		CTV-GTV		CTV_{eval}			Conformity and homogeneity index			
		**Beam 1 [°]**	**Beam 2 [°]**	**Beam 3 [°]**	**$D_{95\%}$ [%]**	**$D_{2\%}$ [%]**	**D_{mean} [Gy]**	**$D_{95\%}$ [%]**	**$D_{95\%}$ [%]**	**$D_{2\%}$ [%]**	**D_{mean} [Gy]**	**$D_{95\%}$ [%]**	**$D_{2\%}$ [%]**	**GTV CI [ ]**	**GTV HI [ ]**	**CTV CI [ ]**	**CTV_{eval} HI [ ]**
**(S3)**	**1**	135/0	220/0	270/20	97.3	103.2	52.8	97.5	97.4	128.6	50.5	97.1	101.4	0.89	5.37	0.67	3.69
	**2**	150/0	200/0	270/20	97.6	103.3	55.7	98.0	97.7	130.2	50.6	97.1	102.2	0.63	5.08	0.70	4.55
	**3**	150/0	200/0	270/25	97.0	103.5	57.6	99.1	98.4	130.0	50.5	97.6	101.5	0.58	5.88	0.69	3.48
	**4**	155/0	210/0	270/20	96.9	102.7	54.1	99.4	99.0	126.0	51.5	98.7	103.7	0.66	5.37	0.73	4.33
	**5**	160/0	220/0	270/15	98.3	102.4	52.4	97.2	97.1	128.2	50.8	96.9	103.7	0.75	3.66	0.83	5.46
	**6**	170/0	215/0	270/10	98.3	102.2	56.7	99.3	99.0	130.8	50.9	98.4	101.8	0.79	3.48	0.54	2.87
**Median**					**97.4**	**102.9**	**54.9**	**98.5**	**98.1**	**129.3**	**50.7**	**97.3**	**102.0**	**0.70**	**5.18**	**0.70**	**4.01**
**(S4)**	**1**	135/10	195/315	245/15	97.7	103.0	52.8	97.7	96.8	128.4	50.6	97.3	101.6	0.88	4.75	0.66	3.59
	**2**	150/335	200/315	250/25	97.8	102.9	55.7	98.1	97.8	130.0	50.7	97.2	102.2	0.64	4.60	0.69	4.40
	**3**	150/350	210/305	250/25	98.5	102.5	59.8	99.4	98.8	131.1	50.7	97.6	101.8	0.57	3.37	0.59	3.61
	**4**	170/345	220/320	260/15	97.0	102.4	54.2	99.8	99.5	126.0	51.5	99.1	103.4	0.68	5.03	0.72	3.66
	**5**	150/355	210/310	260/10	98.4	102.4	52.3	96.5	96.5	128.2	50.6	96.2	103.1	0.75	3.52	0.86	5.63
	**6**	175/340	215/315	270/10	98.3	102.3	56.6	99.3	98.9	130.2	50.9	98.4	101.8	0.59	3.52	0.55	2.78
**Median**					**98.0**	**102.4**	**55.2**	**98.7**	**98.1**	**129.2**	**50.7**	**97.5**	**102.0**	**0.66**	**4.06**	**0.67**	**3.64**

Abbreviations: D_{mean}: mean dose; $D_{2\%}$: near dose maximum; dose received by 2% of the volume; $D_{X\%}$: dose received by x% of the volume
