# Supplementary material for: Comparison of different treatment planning approaches for intensity-modulated proton therapy with simultaneous integrated boost for pancreatic cancer
Source: Radiat Oncol. 2018 Nov 22;13:228. doi: 10.1186/s13014-018-1165-0 (PMC6249773; doi:10.1186/s13014-018-1165-0)
Supplement: Supplementary file 3 — Statistical analysis. (PDF 511 kb) [file 13014_2018_1165_MOESM3_ESM.pdf]

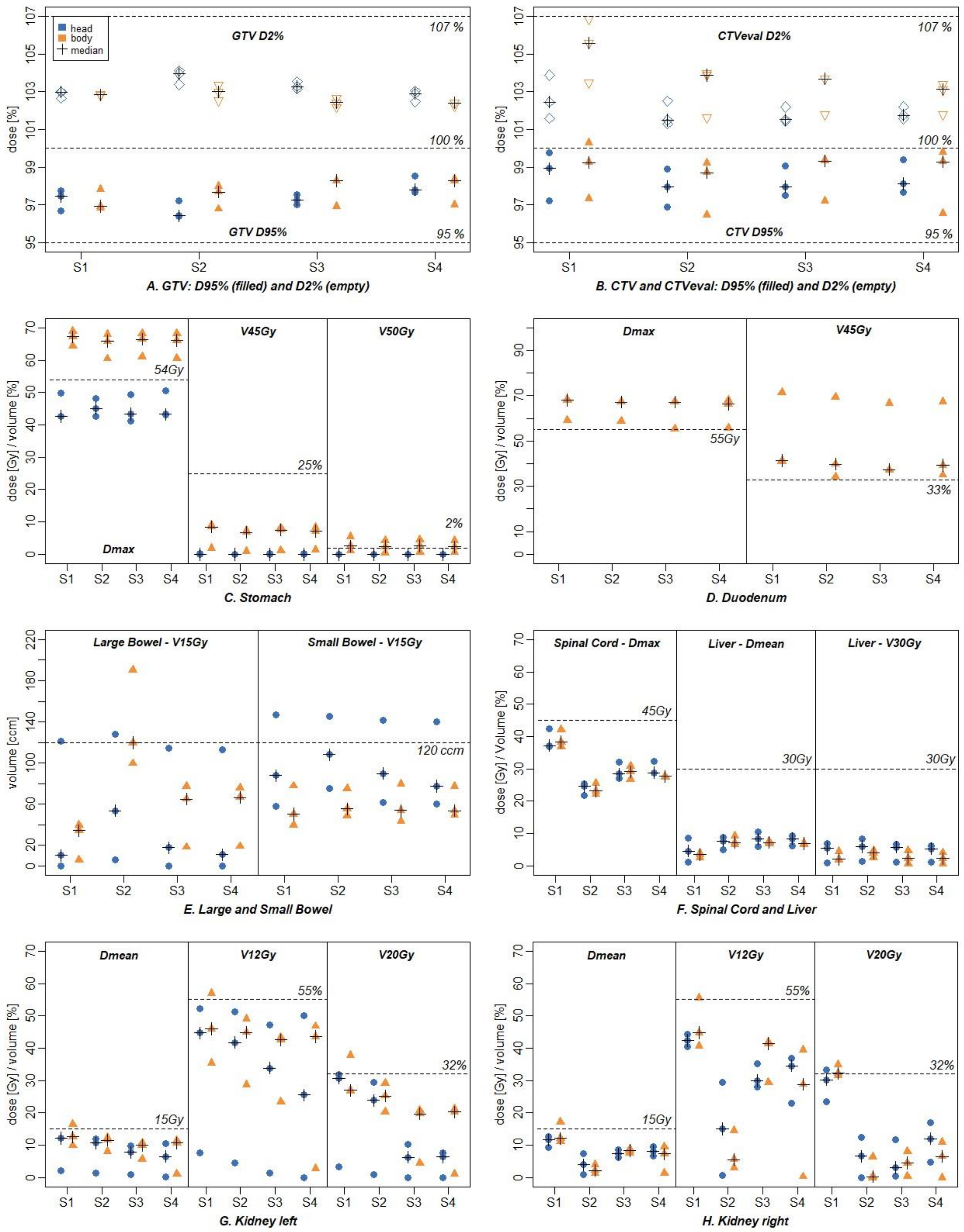

Additional file 3: Dose parameters for the target volumes (A, B) and the organs at risk (C-F) with median values (black crosses) sorted by the four different treatment planning strategies and the location of the tumor (blue: pancreatic head; orange: pancreatic body). Dose constraints are marked with dashed lines. (Abbreviations: D_{mean}: mean dose; D_{max}: maximum dose; V_{xGy}: volume receiving x-Gy; $D_{2\%}$: near dose maximum, dose received by 2% of the volume; $D_{95\%}$: dose received by 95% of the volume).
