# Supplementary material for: Comparison of different treatment planning approaches for intensity-modulated proton therapy with simultaneous integrated boost for pancreatic cancer
Source: Radiat Oncol. 2018 Nov 22;13:228. doi: 10.1186/s13014-018-1165-0 (PMC6249773; doi:10.1186/s13014-018-1165-0)
Supplement: Supplementary file 4 — Subgroup analysis. (PDF 487 kb) [file 13014_2018_1165_MOESM4_ESM.pdf]

**Additional file 4:** *Friedman test* for dependent, not normally distributed variables with the post hoc test of *Dunn* with a *Bonferroni* correction.

| | | **Friedman test (α=0.05)** | | | **Dunn test (α=0.05)** | | | | | |
|---|---|---|---|---|---|---|---|---|---|---|
| **Dose parameter** | | **Chi$^2$** | **df** | ***p*-value** | ***p*-value** | | | | | |
| | | | | | ***S1-S2*** | ***S1-S3*** | ***S1-S4*** | ***S2-S3*** | ***S2-S4*** | ***S3-S4*** |
| GTV | $D_{95\%}$ | 14.3 | 3 | **0.003** | 1.000 | 1.000 | 0.113 | 0.113 | **0.002** | 1.000 |
| | $D_{2\%}$ | 10.2 | 3 | **0.017** | 0.265 | 1.000 | 1.000 | 0.265 | **0.010** | 1.000 |
| | CI | 6.5 | 3 | 0.090 | - | - | - | - | - | - |
| | HI | 13.6 | 3 | **0.004** | 1.000 | 1.000 | 0.152 | 0.152 | **0.002** | 1.000 |
| CTV | $D_{95\%}$ | 10.8 | 3 | **0.013** | **0.015** | 1.000 | 1.000 | 0.265 | 0.061 | 1.000 |
| | CI | 6.2 | 3 | 0.102 | - | - | - | - | - | - |
| CTV-GTV | $D_{mean}$ | 6.2 | 3 | 0.104 | - | - | - | - | - | - |
| | $D_{95\%}$ | 11.6 | 3 | **0.009** | **0.010** | 1.000 | 1.000 | 0.152 | **0.044** | 1.000 |
| | $D_{2\%}$ | 3.0 | 3 | 0.392 | - | - | - | - | - | - |
| $CTV_{eval}$ | $D_{mean}$ | 11.4 | 3 | **0.010** | **0.005** | 0.705 | 1.000 | 0.442 | 0.265 | 1.000 |
| | $D_{95\%}$ | 11.6 | 3 | **0.009** | **0.044** | 1.000 | 1.000 | 0.152 | **0.010** | 1.000 |
| | $D_{2\%}$ | 11.4 | 3 | **0.010** | 0.083 | **0.022** | **0.044** | 1.000 | 1.000 | 1.000 |
| | HI | 12.0 | 3 | **0.007** | 1.000 | **0.044** | **0.010** | 1.000 | 0.442 | 1.000 |
| Left kidney | $D_{mean}$ | 16.2 | 3 | **0.001** | 1.000 | **0.005** | **0.005** | 0.265 | 0.265 | 1.000 |
| | $V_{12Gy}$ | 12.0 | 3 | **0.007** | 1.000 | **0.010** | **0.044** | 0.442 | 1.000 | 1.000 |
| | $V_{20Gy}$ | 16.5 | 3 | **0.001** | 1.000 | **0.003** | **0.007** | 0.202 | 0.344 | 1.000 |
| Right kidney | $D_{mean}$ | 16.2 | 3 | **0.001** | **0.000** | 0.265 | 0.265 | 0.265 | 0.265 | 1.000 |
| | $V_{12Gy}$ | 13.6 | 3 | **0.004** | **0.002** | 1.000 | 0.152 | 0.152 | 1.000 | 1.000 |
| | $V_{20Gy}$ | 14.4 | 3 | **0.002** | **0.003** | **0.022** | 0.561 | 1.000 | 0.442 | 1.000 |

| | | | | | | | | | | |
|---|---|---|---|---|---|---|---|---|---|---|
| Spinal cord | $D_{max}$ | 16.2 | 3 | **0.001** | **0.000** | 0.265 | 0.265 | 0.265 | 0.265 | 1.000 |
| | $D_{2\%}$ | 14.6 | 3 | **0.002** | **0.001** | 0.442 | 0.705 | 0.265 | 0.152 | 1.000 |
| Liver | $D_{mean}$ | 12.2 | 3 | **0.007** | 0.265 | **0.010** | **0.022** | 1.000 | 1.000 | 1.000 |
| | $V_{30Gy}$ | 13.5 | 3 | **0.004** | 0.061 | 1.000 | 1.000 | 0.705 | **0.003** | 0.344 |
| Stomach | $D_{max}$ | 3.7 | 3 | 0.294 | - | - | - | - | - | - |
| | $D_{2\%}$ | 1.8 | 3 | 0.619 | - | - | - | - | - | - |
| | $V_{45Gy}$ * | 8.4 | 3 | **0.038** | 0.152 | 1.000 | 1.000 | 1.000 | 0.442 | 1.000 |
| | $V_{50Gy}$ | 4.3 | 3 | 0.228 | - | - | - | - | - | - |
| Small bowel | $V_{15Gy}$ | 3.8 | 3 | 0.284 | - | - | - | - | - | - |
| Large bowel | $V_{15Gy}$ | 13.1 | 3 | **0.004** | **0.005** | 1.000 | 1.000 | 0.152 | 1.000 | 0.083 |
| | **No differences were shown by the post hoc test due to the multiple testing correction of Bonferroni.* | | | | | | | | | |
