# Supplementary material for: Comparison of different treatment planning approaches for intensity-modulated proton therapy with simultaneous integrated boost for pancreatic cancer
Source: Radiat Oncol. 2018 Nov 22;13:228. doi: 10.1186/s13014-018-1165-0 (PMC6249773; doi:10.1186/s13014-018-1165-0)
Supplement: Supplementary file 5 — Robustness evaluation. (PDF 415 kb) [file 13014_2018_1165_MOESM5_ESM.pdf]

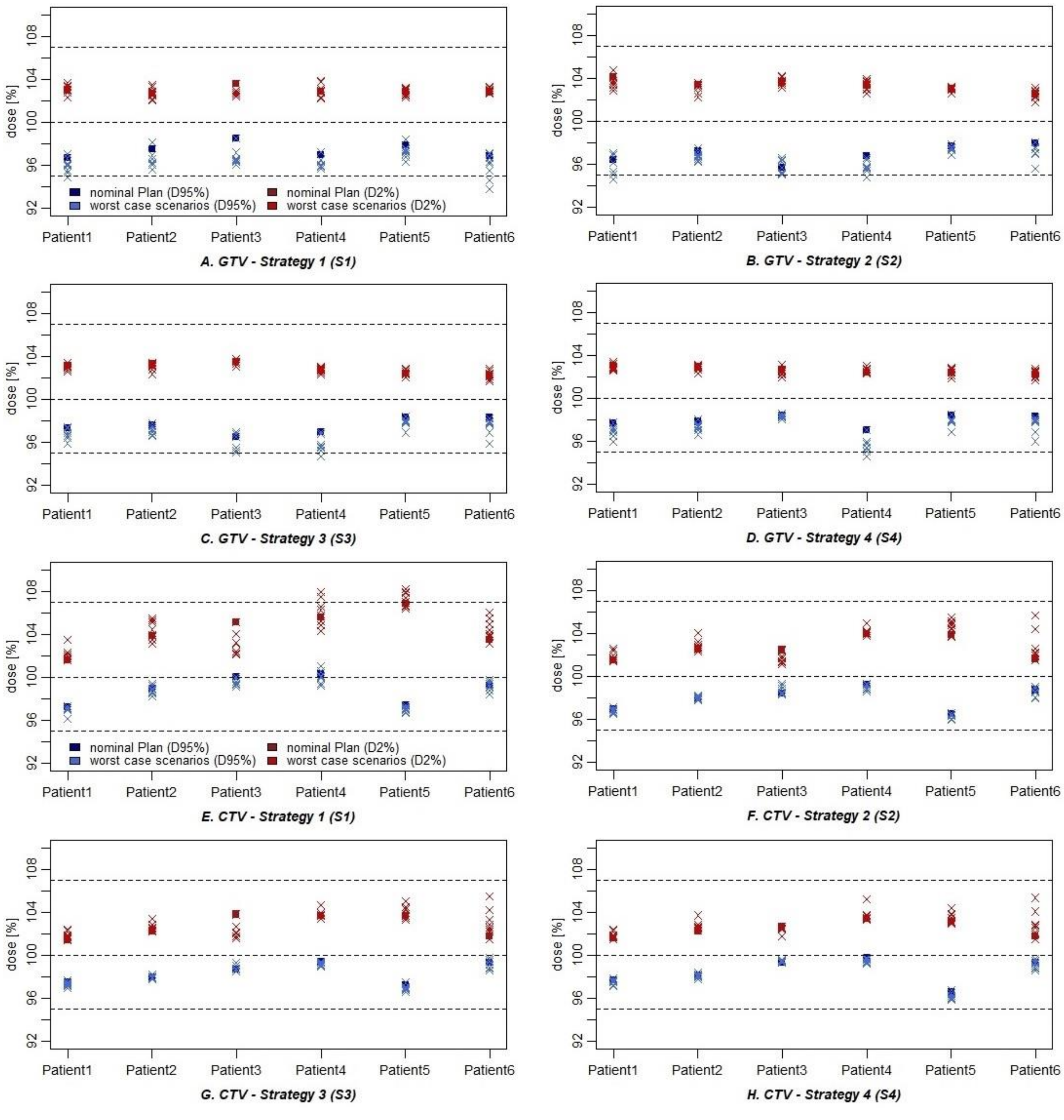

Additional file 5: The robustness analysis of the dose coverage ($D_{95\%}$ and $D_{2\%}$). In this figure the nominal value (square) with the values of nine possible scenarios (cross) for the GTV (A-D) and the CTV (E-H; $D_{95\%}$:CTV, $D_{2\%}$: CTV_{eval}) are shown. It has to be mentioned, that the robust optimization algorithm is influenced by the choice of each optimization objective and their weight. (Abbreviations: $D_{2\%}$: near dose maximum, dose received by 2% of the volume; $D_{95\%}$: dose received by 95% of the volume).
